# *Colletotrichum* Spp. Diversity Between Leaf Anthracnose and Crown Rot From the Same Strawberry Plant

**DOI:** 10.3389/fmicb.2022.860694

**Published:** 2022-04-14

**Authors:** Shuodan Hu, Yanting Zhang, Hong Yu, Jiayan Zhou, Meihua Hu, Aichun Liu, Jianyan Wu, Hancheng Wang, Chuanqing Zhang

**Affiliations:** ^1^College of Modern Agriculture, Zhejiang Agriculture and Forestry University, Hangzhou, China; ^2^Research Institute for the Agriculture Science of Hangzhou, Hangzhou, China; ^3^Agricultural Technology Extension Center of Zhejiang Province, Hangzhou, China; ^4^Guizhou Academy of Tobacco Science, Guiyang, China

**Keywords:** strawberry, *Colletotrichum* spp., crown rot, leaf anthracnose, biological characteristics, pathogenicity

## Abstract

Leaf anthracnose (LA) and anthracnose crown rot (ACR) represent serious fungal diseases that pose significant threats to strawberry production. To characterize the pathogen diversity associated with above diseases, 100 strawberry plants, including varieties of “Hongjia,” “Zhangji,” and “Tianxianzui,” were sampled from Jiande and Zhoushan, the primary plantation regions of Zhejiang province, China. A total of 309 *Colletotrichum* isolates were isolated from crown (150 isolates) and leaves (159 isolates) of affected samples. Among these, 100 isolates obtained from the plants showing both LA and CR symptoms were selected randomly for further characterization. Based on the morphological observations combined with phylogenetic analysis of multiple genes (*ACT*, ITS, *CAL*, *GAPDH*, and *CHS*), all the 100 tested isolates were identified as *C. gloeosporioides* species complex, including 91 isolates of *C*. *siamense*, 8 isolates of *C*. *fructicola* causing both LA and ACR, and one isolate of *C*. *aenigma* causing ACR. The phenotypic characteristics of these isolated species were investigated using the BIOLOG phenotype MicroArray (PM) and a total of 950 different metabolic phenotype were tested, showing the characteristics among these isolates and providing the theoretical basis for pathogenic biochemistry and metabolism. The pathogenicity tests showed that even the same *Colletotrichum* species isolated from different diseased tissues (leaves or crowns) had significantly different pathogenicity toward strawberry leaves and crown. *C. siamense* isolated from diseased leaves (CSLA) was more aggressive than *C. siamense* isolated from rotted crown (CSCR) during the infection on “Zhangji” leaves. Additionally, *C. fructicola* isolated from affected leaf (CFLA) caused more severe symptoms on the leaves of four strawberry varieties compared to *C. fructicola* isolated from diseased crown (CFCR). For crown rot, the pathogenicity of CSCR was higher than that of CSLA.

## Introduction

Strawberry (*Fragaria* × *ananassa* Duch.) is the most extensively cultivated berry in the world. In recent years, the occurrence of strawberry diseases become increasingly serious, especially in the wet and rainy season (Henz et al., 1992). Strawberry leaf anthracnose (LA) and anthracnose crown rot (ACR) are important fungal diseases that mainly occur during both the nursery and colonization periods of strawberries worldwide (Freeman and Katan, 1997; Freeman et al., 2001; Curry et al., 2002). *Colletotrichum* spp. as the causing agents of the above diseases are able to cause irregular circular spots on leaves (Karimi et al., 2019), invade the shortened crown and expands into an annular ring, making strawberry seedlings unable to absorb water and nutrients and killing seedlings (Paynter et al., 2016).

*Colletotrichum gloeosporioides*, *C*. *acutatum*, and *C*. *fragariae* are the main causing agents of LA in strawberry plants (Freeman et al., 1998). Compared to LA, crown rot (CR) is more complicated. At present, as many as about 20 different species of pathogens have been reported in the main strawberry-producing areas, including *C*. *gloeosporioides* (Jacobs et al., 2020), *Fusarium oxysporum* (Juber et al., 2014), and *Phytophthora* spp. (Li et al., 2011). In the past, LA and ACR on strawberries were considered to be caused by the same *Colletotrichum* species, namely: *C*. *acutatum*, *C*. *fragariae*, and *C*. *gloeosporioides* caused similar disease symptoms on the crowns and leaves of strawberry (Freeman and Rodriguez, 1994). *C*. *acutatum* has been found on stubs, petioles, and fruits in most areas of the US and on runner stubs, petioles, and leaves of plants in Florida. However, it was also hypothesized that strawberry LA and ACR in Florida was cause by different *Colletotrichum* spp. (Howard et al., 1992). *C*. *fragariae* cause all symptoms, except leaf spots, and *C*. *acutatum* causes all symptoms, except ACR (Howard et al., 1992). Interestingly, previous researchers also speculated that different *Colletotrichum* spp. are specific to the organ of infection, even though they can cause LA and ACR. *C*. *gloeosporioides* and *C*. *fragariae* usually infect petioles, stolons, and the crown, but they occasionally cause fruit rot (Peres et al., 2005). *C*. *acutatum* (species complex) often infects petiols and the crown as well as fruits (Peres et al., 2005). Both species can cause both LA and ACR, however, *C*. *gloeosporioides* is most often associated with ACR, while *C*. *acutatum* is considered the main LA pathogen (Ureña-Padilla et al., 2002). According to the survey on strawberry diseases conducted by Zhong et al. (2020), three species of *Colletotrichum*, *C. fructicola*, *C. siamense*, and *C. nymphaeae* were isolated and identified to infect different strawberry organs. *C*. *siamense* showed strong aggressiveness toward fruit and leaves. *C*. *fructicola* showed strong aggressiveness toward the crown. *C*. *siamense* showed more pathogenic than *C*. *fructicola* or *C*. *nymphaeae*. The difference in pathogenicity may be related to metabolic phenotype characterization (Wang et al., 2015). In 2019, our previous study showed that all pathogenic fungi on strawberry plants belong to the *C*. *gloeosporioides* species complex in Zhejiang (Chen et al., 2020).

The classification and identification of *Colletotrichum* spp. according to morphological characteristics is difficult because of their instability (Cannon et al., 2012). Phylogenetic analysis based on internal transcribed spacer (ITS) rDNA may also be uncertain (Xie et al., 2010). At present, combining multi-gene phylogenetic analysis with morphological characteristics is being used to identify many important pathogens (Cai et al., 2009). Wang et al. (2019) combined ITS, *TUB2*, *GAPDH*, and *CHS1* with morphology to confirm that the species of pathogenic fungi causing fruit and crown rot was the *C*. *acutatum* species complex. Bi et al. (2017) combined multi-gene phylogenetic analysis with morphological characteristics to verify that *C*. *truncatum* was the species causing LA and fruit rot. However, analysis and comparison of *Colletotrichum* spp. causing LA and ACR on strawberries in China has not been performed. In this study, we first identified the LA and ACR causing agents using morphological characterization and phylogenetic analysis of multiple genes (*ACT*, ITS, *CAL*, *GAPDH*, and *CHS*), and the isolates were further tested for metabolic phenotype and pathogenicity analysis to compare the different biological and pathogenic characteristics among *Colletotrichum* spp.

## Materials and Methods

### Disease Symptoms, Sampling, and Fungal Isolation

For diseased sample collections, we sampled three strawberry varieties including “Hongjia,” “Zhangji,” and “Tianxianzui.” For each variety, ten strawberry plants were randomly sampled from two greenhouses. The strawberry varieties “Hongjia” and “Zhangji” were collected from four greenhouses in Zhoushan, varieties “Hongjia,” “Zhangji,” and “Tianxianzui” were collected from six greenhouses in Jiande. A total of 100 strawberry plants were collected from 10 greenhouses in the main growing areas of Zhejiang Province (27°02′ to 31°11′ N, 118°01′ to 123°10′ E), China.

For *Colletotrichum* spp. isolation, the diseased tissues were washed with running tap water, cut into 5 × 5 mm pieces using sterilized scissors, soaked in 75% alcohol for 30 s, and then soaked in 3% sodium hypochlorite solution for 2 min, washed with sterile distilled water three times, and dried on sterile filter paper. Each tissue piece was placed on a plate containing potato dextrose agar (PDA) medium supplemented with kanamycin sulfate and streptomycin sulfate (100 mg/L) and incubated at 25°C. After 3 to 5 days incubation, the corresponding mycelia were then transferred to a new PDA plate (Phoulivong et al., 2010; Noman et al., 2018). The single-conidium isolates were stored on PDA slants at 4°C.

### Morphological Characterization

Morphological and cultural characterizations were performed according to the methods described by Cai et al. (2009). Briefly, the mycelia plugs (5 mm in diameter) taken from the margin of 5-day-old colony of tested isolates were transferred to the new PDA plates and incubated at 25°C. The experiment was designed as a randomized complete block, with three replicates for each isolate. After 7 days, the colony diameter was measured, and the growth rate was calculated; the colony color of the aerial hyphae and morphology were recorded as previously described (Cai et al., 2009; Weir et al., 2012).

Conidial suspensions of each isolate were prepared in lactic acid from conidial masses on PDA. The shape and color of the conidia were observed, and the sizes of 50 conidia from each isolate were measured at 400× magnification under a light microscope (Carl Zeiss Microscopy GmbH, Gottingen, Germany). The sporulation and germination rates of each isolate were calculated as previously described (Johnny et al., 2011).

### Molecular Identification and Phylogenetic Analysis

The total DNA of each tested isolate was extracted using the fungi genomic DNA rapid extraction kit (B518229-0100; Sangon Biotech). The target genes selected in this study were actin (*ACT*), calmodulin (*CAL*), chitin synthase (*CHS*), glyceraldehyde 3-phosphate dehydrogenase (*GAPDH*), and internal transcribed spacer (ITS). The cycling parameters and primers to amplify *ACT*, *CAL*, *CHS*, *GAPDH*, and ITS used in this study were the same as those described by Damm et al. (2012) and Weir et al. (2012). The primers selected for corresponding PCR amplification are shown in Table 1. The PCR products from a 50 μL amplification system were verified on 1.0% agarose gel at 254 nm (UV) and further sequenced by Shanghai Sangon Biological.

**TABLE 1:** Primers used in this study with sequences and sources.

Gene	Primers	Direction	Length (bp)	Sequence	References
ACT^z^	ACT-512F	Forward	399	ATGTGCAAGGCCGGTTTCGC	Carbone and Kohn, 1999
	ACT-783R	Reverse		TACGAGTCCTTCTGGCCCAT	Carbone and Kohn, 1999
CAL	CL1C	Forward	773	GAATTCAAGGAGGCCTTCTC	O’Donnell et al., 2000
	CL2C	Reverse		CTTCTGCATCATGAGCTGGAC	O’Donnell et al., 2000
CHS-I	CHS-79F	Forward	779	TGGGGCAAGGATGCTTGGAAGAAG	Carbone and Kohn, 1999
	CHS-354R	Reverse		TGGAAGAACCATCTGTGAGAGTTG	Carbone and Kohn, 1999
GAPDH	GDF	Forward	870	GCCGTCAACGACCCCTTCATTGA	Templeton et al., 1992
	FDR	Reverse		GGGTGGAGTCGTACTTGAGCATGT	Templeton et al., 1992
ITS	Its-1F	Forward	593	CTTGGTCATTTAGAGGAAGTAA	Gardes and Bruns, 1993
	Its-4	Reverse		TCCTCCGCTTATTGATATGC	White et al., 1990

*^z^ATC, actin; CAL, calmodulin; CHS-1, chitin synthase 1; GAPDH, glyceraldehyde 3-phosphate dehydrogenase; ITS, internal transcribed spacer.*

All sequences were grouped and aligned using DNAMAN v. 7.0. Sequences with single nucleotide polymorphisms were amplified and sequenced three times to ensure that the observed polymorphisms were not due to sequencing errors. The 100 tested isolates and referenced standard isolates (Weir et al., 2012; Diao et al., 2017) used in this study are listed in Supplementary Table 1. *C*. *boninense* (CBS: 123755) was used as the outgroup (Weir et al., 2012). All sequences were aligned using Clustal X 2.0.10 (Larkin et al., 2007). Gene sequences were compared and corrected using the “W” function in MEGA 5.0 (Tamura et al., 2011). The gene sequences were concatenated. Modeltest3.7.win, Win-paup4b10-console, and Mrmodeltest2, as implemented in MrMTgui, were used to estimate the best model of nucleotide substitution (Vaidya et al., 2011). Bayesian inference (BI) phylogenies were constructed using Mr. Bayes v. 3.1.2 (Ronquist and Huelsenbeck, 2003). Six simultaneous Markov chains were run for 1,000,000 generations each, and trees were sampled every 100th generation. The first 2,000 trees, representing the burn-in phase of the analyses, were discarded, and the remaining 8,000 trees were used to calculate the posterior probability (PP) in the majority rule consensus tree. Phylogenetic trees were drawn using treeView (Page, 1996). The alignments and trees were deposited in treeBase.

### Pathogenicity Testing

Each of the 100 tested isolate was cultured on a PDA medium for 5 days at 25°C. The spore suspensions were prepared following Gale et al. (2005). Five hyphae blocks (diameter of 5 mm) taken from the colony margin were transferred to 100 mL PD liquid medium and shaken at 160 r/min and 25°C to induce conidia production. After 5 days, spores were collected and adjusted to 1.0 × 10^6^ conidia/mL using a hemocytometer.

Strawberry seedlings (“Hongjia,” “Zhangji,” and “Tianxianzui” varieties) were planted in pots filled with sterilized nutrient soil and placed at 25 ± 5 °C under 12 h light and 12 h darkness. Five-week-old healthy strawberry plants had not been treated with any chemicals, were adopted for inoculation. Each leaf was inoculated with 10 μL spore suspension drop, and each crown was sprayed with 30 mL of the spore suspension. The negative control was inoculated with sterile water, three plants were inoculated for each isolate and all 100 isolates were tested. The plants were then placed back in a greenhouse at 25 ± 5°C with 90% humidity for 7 days under a 12 h light/dark cycle. The pathogens were re-isolated from the diseased leaves or crowns to complete Koch’s postulates (Chen et al., 2020).

### Comparison of Pathogenicity Differences

We randomly selected representative isolates to determine the pathogenicity on leaves (“Hongjia,” “Zhangji,” “Tianxinazui,” “Hongyu”) and the crown (“Hongjia,” “Zhangji,”, “Tianxinazui”). These included, JD-J-ZJ-3-2, JD-J-ZJ-5, and JD-J-ZJ-9 from *C*. *fructicola* obtained from crown rot (CFCR), ZS-J-ZJ-54, ZS-J-ZJ-111, and JD-J-HX-A-6 from *C*. *siamense* obtained from crown rot (CSCR), JD-HX-Y-H-22, JD-ZJ-Y-H-69, and ZS-ZJ-Y-2-2 from *C*. *fructicola* obtained from leaf anthracnose (CFLA), JD-HX-Y-B-7, JD-ZJ-Y-H-60, and ZS-HX-Y-8 from *C*. *siamense* obtained from leaf anthracnose (CSLA) were adopted.

The spore suspension was prepared, and the leaves and crown were inoculated according to the methods described above. Both wounded and non-wounded plants were inoculated, the wounded condition using pin-pricking method (Karimi et al., 2019). Depending on the size of the leaf, using a sterile sharp needle pricking 4–12 wounds on the adaxial surface of each leaf. Before inoculation, leaves were surface sterilized by dipping in 1% sodium hypochlorite for 30 s and ethanol 70% followed by washed three times with distilled water, and then left to dry on sterile paper. For crown, using sterilizing blades caused several wounds at the crown. For each isolate, the inoculation was repeated three times with three leaves and crowns for each repeat. The diameter of the lesions on the leaves was measured 7 days after inoculation (Han et al., 2016). The severity standard of crown rot was recorded following (Haack et al., 2018). One-way ANOVA was performed to analyze the significance difference using IBM SPSS Statistics V22 software, and the least significant difference (LSD) test was applied to separated mean values for different species in the pathogenicity test at *P* = 0.05 level.

### Phenotype Characterization

Three *Colletotrichum* spp. were characterized to determine the phenotype using the Biolog standard procedure (Bochner et al., 2001; Bochner, 2003; von Eiff et al., 2006). The concentration of the spore suspension was adjusted to 1 × 10^6^ conidia/mL. Metabolic plates 1–10 were used to assess the metabolic pathways of carbon (PM1–2), nitrogen (PM3), phosphorus and sulfur (PM4), bio-synthetic pathways (PM5), peptide nitrogen (PM 6-8), osmotic and ionic conditions (PM9), and pH conditions (PM10). To each well of the PM plates, 100 μL of a cell suspension was added with 62% transmittance. The metabolic phenotype test plate was placed in the Biolog system incubator at 28°C for 7 days. Phenotypic data were recorded every 15 min by the OmniLog 2.4 system. Heatmaps of phenotype analysis were generated with HemI software (Heatmap Illustrator, version 1.0; Zhao et al., 2017).

## Results

### Fungal Isolates of Pathogenic *Colletotrichum* spp. From Strawberries

A total of 309 *Colletotrichum* spp. were isolated, with 150 from the crown and 159 from leaves. Pathogenicity was determined according to the Koch’s postulate, and all isolates were pathogenic. We selected 50 strawberry plants at random, from which *Colletotrichum* spp. were successfully isolated from both the crown and leaves. In Jiande, 10, 10, and 9 plants were selected for “Hongjia,” “Zhangji,” and “Tianxianzui,” respectively. In Zhoushan, 11 and 10 plants were selected for “Hongjia” and “Zhangji,” respectively. A total of 100 isolates were collected for further characterization, including 50 for LA and 50 for CR.

**FIGURE 1:**
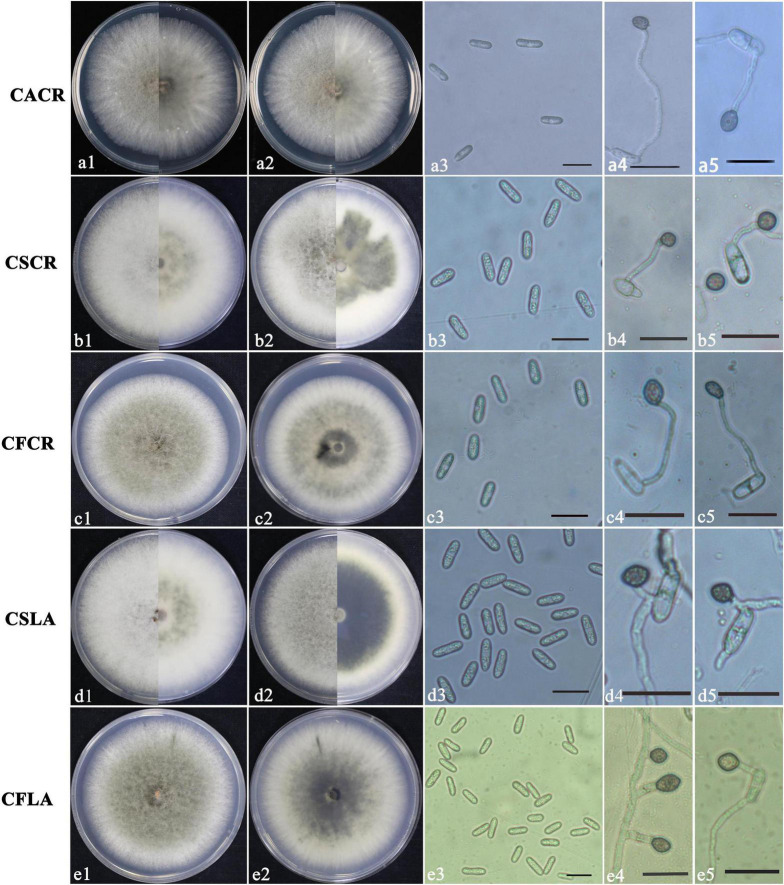
Colony morphology of CACR (*Colletotrichum aenigma* causing crown rot), CSCR (*C. siamense* causing crown rot), CSLA (*C. siamense* causing leaf anthracnose), CFCR (C. *fructicola* causing crown rot) and CFLA (*C. fructicola* causing leaf anthracnose) on PDA **(a1–e1,a2–e2)**, conidia **(a3–e3)** and appressoria **(a4–e4,a5–e5)**, scale bar in 3 = 20 μm in **(a3–e3**,**a4–e4**,**a5–e5)**.

According to morphological and cultural characterizations, all the 100 isolates were grouped into three *Colletotrichum* species. The colony morphology of the above three *Colletotrichum* species on PDA media did not differ substantially (Figure 1). *C*. *fructicola* produced a gray-white colony with irregular edges and colony was black on the back on PDA plates; the conidium heap was orange, and conidia were cylindrical, with both ends obtuse or pointed. Appressoria were single or scattered, and most were light brown to dark brown, spherical, cylindrical, or fusiform, rare for irregular type; a few produced two suborbicular appressoria. The *C*. *siamense* colony was fluffy, gray in the middle and white at the edge, neat, yellow in the middle on the back, and white at the edge. The conidia were dark yellow, straight, cylindrical, blunt and round at both ends, colorless, and smooth. Appressoria were brown, and/or oval or spindle-shaped, with complete edges, and a few irregularities. The aerial hyphae of *C*. *aenigma* were sparse, light gray to white. The colony was pale white on the back and gray–green in the center. Conidia heaps were rare, and the conidia were stick-like, blunt, and round at both ends, single, or loosely distributed; appressoria were dark brown and ovate or irregularly shaped.

The significant morphological differences among three *Colletotrichum* spp. were listed in Table 2. *C*. *fructicola* had the largest spores, and *C*. *aenigma* had the largest appressoria. *C*. *siamense* had a higher growth rate, sporulation ability, and spore germination rate than that of *C*. *fructicola* and *C*. *aenigma*.

**TABLE 2:** Size of spore, appresoria, hyphae growth rates, sporulation and spore germination rate of different *Colletotrichum* species from strawberry.

Species	Characteristics^x^	Conidia^y^		Appresoria^y^		Growth rate (mm/day)^y^	Sporulation (×10^6^)^y^	Germination rate (%)^y^
		Length (μm)	Width (μm)	Length (μm)	Width (μm)			
*C. fructicola*	Off White, with	17.69 ± 0.22 a	7.21 ± 0.17 a	8.55 ± 0.13 b	6.81 ± 0.10 a	13.71 ± 0.11 b	18.03 ± 3.93 a	15.92 ± 2.60 b
*C. siamense*	Off White, with	16.28 ± 0.32 b	7.14 ± 0.20 a	7.23 ± 0.10 c	6.35 ± 0.08 b	14.22 ± 0.05 a	21.22 ± 3.07 a	24.94 ± 1.67 a
*C. aenigma*	Off White, with	15.03 ± 0.19 c	4.96 ± 0.08 b	9.38 ± 0.30 a	6.66 ± 0.13 ab	13.45 ± 0.10 b	5.62 ± 1.85 b	9.27 ± 1.64 b

*^x^Colony characteristics: off white, with, off white mycelia with mass conidial masses.*

*^y^Data is the mean ± standard error. Mean values with the same letters were not statistically different (P > 0.05) according to the least significant difference (LSD) Test.*

**FIGURE 2:**
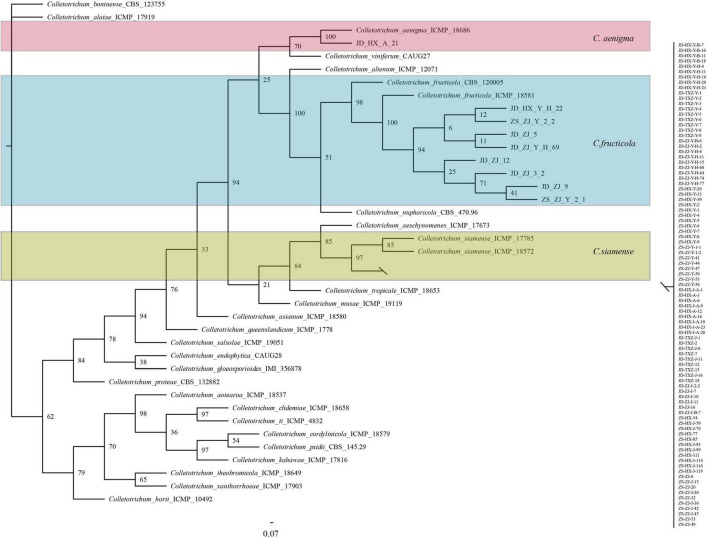
Bayesian inference phylogenetic tree of the *Colletotrichum* spp. isolated from strawberry belonging to *Colletotrichum gloeosporioides* species complex. The tree was constructed based on ACT, CAL, CHS, GAPDH, and ITS. *C. boninense* was used as an outgroup. The scale bar shows 0.07 expected changes per site.

Phylogenetic trees were constructed using combined *ACT*, *GADPH*, *CHS*, *CAL*, and ITS datasets consisting of 100 *Colletotrichum* isolates with *Colletotrichum boninense* (CBC 123755) as the outgroup taxa. The concatenated alignment included 1256 characters. The boundaries of the loci used in the alignment were as follows: *ACT*: 1–152; *CAL*: 153– 448; *CHS*: 449–658; *GADPH*: 659–866; and ITS: 867–1256. All isolates were identified as *C*. *gloeosporioides* species complex and fell into three clades, with 91 isolates clustered in *C*. *siamense*, 8 isolates clustered in *C*. *fructicola*, and one isolate clustered in *C*. *aenigma* (Figure 2).

### Pathogenicity of *Colletotrichum* Species on Strawberry Leaves

**FIGURE 3:**
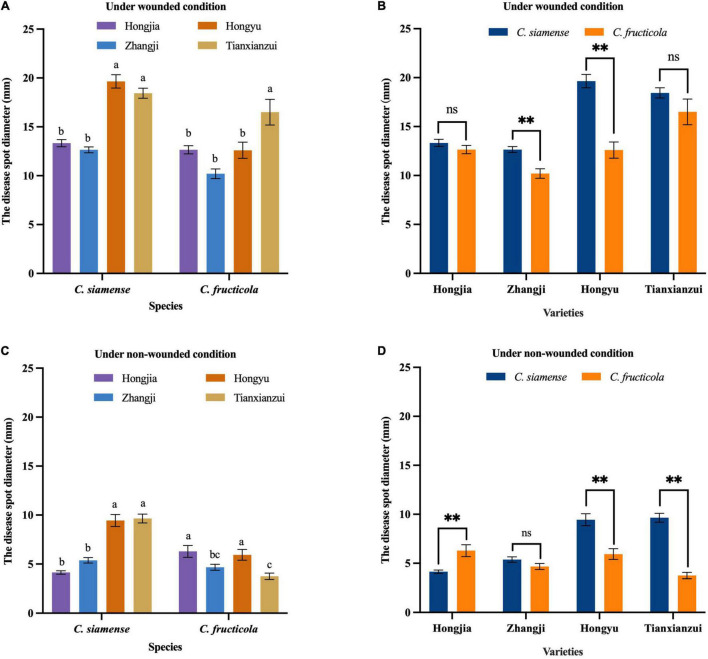
Pathogenicity of *Colletotrichum fructicola* and *C. siamense* to different varieties of leaves. **P = 0.01, ns = not significant. **(A,B)** To strawberry leaves of different varieties under wounded condition. **(C,D)** To strawberry leaves of different varieties under non-wounded condition. Values with the same letters were not statistically different (*P* > 0.05) according to the least significant difference (LSD) Test.

**FIGURE 4:**
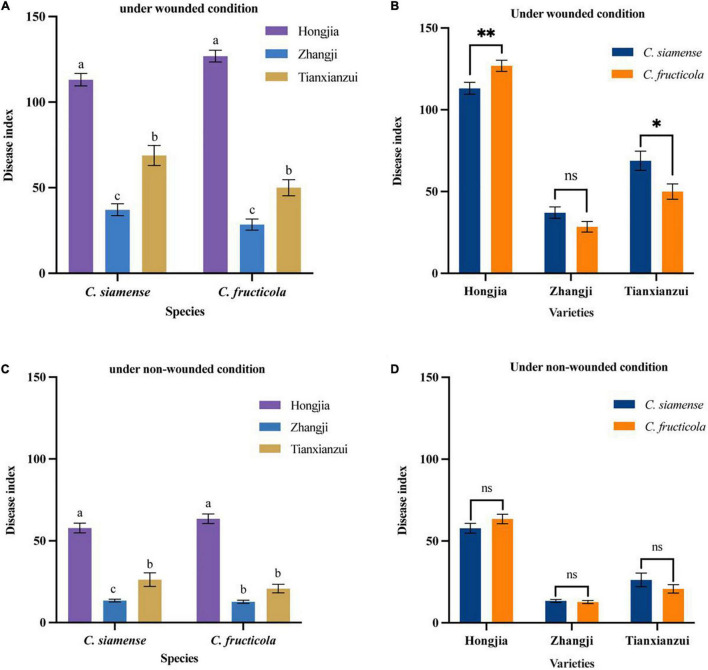
Pathogenicity of *Colletotrichum fructicola* and *C. siamense* to different varieties of crown of strawberry. *P = 0.05, **P = 0.01, ns = not significant. **(A,B)** To strawberry crown of different varieties under wounded condition. **(C,D)** To strawberry crown of different varieties under non-wounded condition. Values with the same letters were not statistically different (*P* > 0.05) according to the least significant difference (LSD) Test.

We conducted the pathogenicity analysis of each isolate on leaves using four varieties: “Hongjia,” “Zhangji,” “Hongyu,” and “Tianxianzui.” Under wounded conditions, *C*. *siamense* and *C*. *fructicola* had significant differences (*P* < 0.01) in the pathogenicity on “Zhangji” and “Hongyu” (Figures 3A,B), and the pathogenicity of *C*. *siamense* was significantly higher than that of *C*. *fructicola*. *C*. *siamense* showed stronger pathogenicity to the “Hongyu” and “Tianxianzui” varieties but weaker pathogenicity to “Hongjia” and “Zhangji” (Figure 4A).

The disease spot diameters on wounded leaves caused by *C*. *siamense* on “Hongyu” and “Tianxianzui” were 19.65 and 18.44 mm, respectively, and on “Hongjia” and “Zhangji,” they were 13.33 and 12.65 mm, respectively. *C*. *fructicola* showed stronger pathogenicity to “Tianxianzui,” with a disease spot diameter of 15.5 mm. Under non-wounded conditions, *C*. *siamense* and *C*. *fructicola* had significantly different pathogenicity indicated by mean disease spot diameters on “Hongjia,” “Hongyu,” and “Tianxianzui” (*P* < 0.01; Figures 3C,D). The pathogenicity of *C*. *siamense* was significantly higher than that of *C*. *fructicola* on “Hongyu” and “Tianxianzui” but weaker than that of *C*. *fructicola* on “Hongjia.”

### Pathogenicity of *Colletotrichum* Species on Strawberry Crown

On the crown, we tested the pathogenicity of three strawberry varieties: “Hongjia,” “Zhangji,” and “Tianxianzui.” Under wounded conditions (Figures 4A,B), the pathogenicity of *C*. *siamense* and *C*. *fructicola* was significantly different on “Hongjia” and “Tianxianzui” but not on “Zhangji.” The pathogenicity of *C*. *siamense* was significantly higher than that of *C*. *fructicola* on “Tianxianzui”; the disease index achieved by *C*. *siamense* and *C*. *fructicola* were 68.83 and 50.00, respectively. However, the pathogenicity of *C*. *siamense* was significantly weaker than that of *C*. *fructicola* on “Hongjia”; the disease index were 113.12 and 126.9, respectively. In general, *C*. *siamense* and *C*. *fructicola* showed the strongest pathogenic ability toward “Hongjia,” followed by “Tianxianzui” and “Zhangji” (Figures 4A,C). There were no significant differences under the non-wounded conditions (Figure 4D).

### Phenotype Characterization of *Colletotrichum* Species From Strawberry

Using PM plates 1–10, three *Colletotrichum* spp. were characterized. Overall, 950 different growth nutrient conditions were tested, including 190 carbon substrates (PM1-2), 95 nitrogen substrates (PM3), 59 phosphorus substrates (PM4), 35 sulfur substrates (PM4), 94 bio-synthetic pathways (PM5), 285 peptide nitrogen substrates (PM6-8), 96 osmotic and ionic conditions (PM9), and 96 pH environments (PM10) (Figure 5).

**FIGURE 5:**
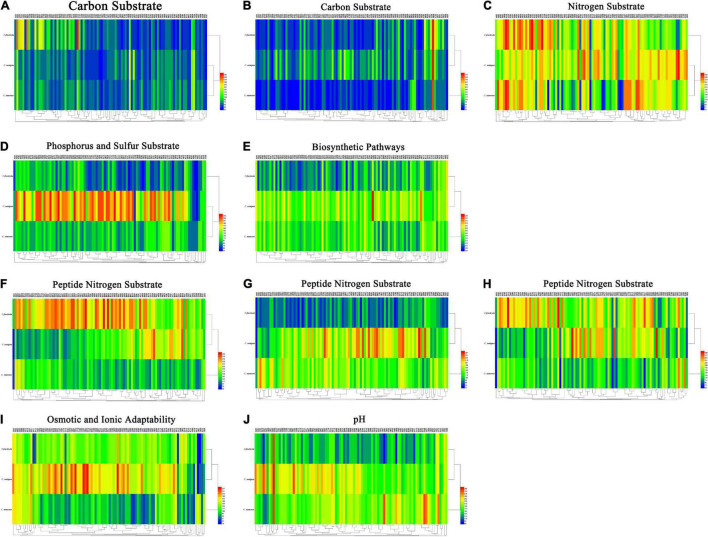
**(A)** Carbon substrate. **(B)** Carbon substrate. **(C)** Nitrogen substrate. **(D)** Phosphorus and sulfur substrate. **(E)** Biosynthetic pathways. **(F)** Peptide nitrogen substrate. **(G)** Peptide nitrogen substrate. **(H)** Peptide nitrogen substrate. **(I)** Osmotic and ionic adaptability. **(J)** pH. Overview of metabolic phenotypes of *Colletotrichum fructicola*, *C. siamense*, *C. aenigma*. Heatmap of maximum area values of sources expressed as maximum curve area monitored during 84 h of incubation. The legend of color code from blue to green, and red shades indicate low, moderate, and high utilization of sources, respectively, assessed as arbitrary Omnilog values.

For carbon source, all three *Colletotrichum* spp. could metabolize 190 carbon sources, such as sugars, nucleotides, and carboxylic acids, and produce color reactions (Figures 5A,B). *C*. *fructicola* had a relatively high availability, especially in L-arabinose (PM1, A02), glycerin (PM1, B03), and dulcitol (PM1, A12), whereas *C*. *siamense* and *C*. *aenigma* had a relatively low availability. The three species showed higher utilization rates with arbutin (PM2, B08) (Figure 5C). For amino acid nitrogen substrate metabolization, *C*. *siamense*, *C*. *fructicola*, and *C*. *aenigma* had high utilization rates. For phosphorus and sulfur substrate metabolization (Figure 5D), *C*. *fructicola* had a relatively high availability, and *C*. *siamense* and *C*. *fructicola* had a relatively low availability. For the bio-synthetic pathways (Figure 5E), C. *aenigma* had the highest overall utilization of nutritional supplement elements, followed by *C*. *siamense* and *C*. *fructicola*. For peptide nitrogen substrate metabolization (Figures 5F–H), *C*. *fructicola* had the best utilization of various peptide nitrogen elements, especially in Ala-Pro (PM6, A12), Arg-Ala (PM6, B05), Arg-Arg (PM6, B06), and Gly-Arg (PM6), E03), and had the highest utilization capacity, followed by *C*. *aenigma* and *C*. *siamense*. For osmotic and ionic adaptability (Figure 5I), *C*. *aenigma* had the highest utilization rate. The three *Colletotrichum* spp. had basically the same metabolic profiles under the condition of 80 mM sodium nitrite (PM9, H11), but the utilization rate was not high. In the pH environmental adaptability tests, *C*. *aenigma* had the highest utilization of pH under different conditions (Figure 5J). The three *Colletotrichum* spp. had basically the same metabolic profile under 5% ethylene glycol (PM10, D09) and showed extremely high utilization rates.

### *Colletotrichum* Species Causing LA and Anthracnose Crown Rot on Strawberry

The LA and ACR in Zhejiang province were caused by different *Colletotrichum* species. Total 50 isolates from LA samples were grouped into two clusters with 8% (4 isolates) as *C*. *fructicola* and 92% (46 isolates) as *C*. *siamense*. For ACR, 4 isolates (8%) were identified as *C*. *fructicola*, 45 isolates (90%) were identified as *C*. *siamense*, and one isolate (2%) was identified as *C*. *aenigma*. Moreover, *C*. *siamense* was the most frequently pathogenic species, causing both LA and ACR in sampled greenhouses.

**FIGURE 6:**
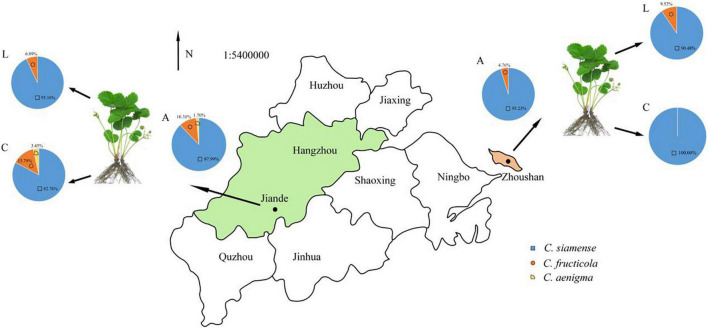
The proportion of *Colletotrichum* spp. associated with LA and CR in Zhejiang province, China. A, all strains; L, leaf strains; C, crown strains, the scale is 1:54,000,000.

In Jiande, *C*. *siamense*, *C*. *fructicola*, and *C*. *aenigma* accounted for 87.99, 10.31, and 1.7% of all collected isolates, respectively. They caused 82.76, 13.79, and 3.45% ACR, and 93.10, 6.89, and 0.0% LA, respectively. In Zhoushan, *C*. *siamense* and *C*. *fructicola* accounted for 95.23 and 4.76% for ACR, 90.48% and 9.52% for LA, respectively (Figure 6).

The size of appressoria and conidia, and the growth rate of hyphae of the five types of *Colletotrichum* spp. are shown in Table 3. The sporulation and germination abilities were significantly different between the same *Colletotrichum* species obtained from plants showing LA or CR symptoms. The sporulation of *C*. *fructicola* causing crown rot (CFCR) was less than *C*. *fructicola* causing leaf anthracnose (CFLA), the *C. siamense* causing crown rot (CSCR) was higher than *C*. *siamense* causing leaf anthracnose (CSLA). The spore germination rate of the CSCR and CFCR was lower than the CSLA and CFLA. However, there was no significant difference (*P* > 0.05) in the size of spores, appressorium size, hyphal growth rate, and sporulation between the same *Colletotrichum* species obtained from plants showing LA and CR symptoms.

**TABLE 3:** Size of spore, appressoria, growth rates, sporulation and spore germination rate of *Colletotrichum* species from leaf and crown of strawberry.

Species^x^	Conidia^y^		Appressori		Growth Rate (mm/day)^y^	Sporulation (×10^6^)	Germination rate (%)^y^
	Length (μm)	Width (μm)	Length (μm)	Width (μm)			
CACR^z^	15.03 ± 0.19 c	4.96 ± 0.08 c	9.38 ± 0.30 a	6.66 ± 0.13 b	13.45 ± 0.10 c	5.62 ± 1.85 b	9.27 ± 1.64 c
CSCR	15.07 ± 0.49 c	6.26 ± 0.26 b	7.09 ± 0.11 d	6.46 ± 0.09 bc	13.51 ± 0.17 bc	22.50 ± 6.76 a	17.48 ± 0.98 b
CSLA	17.58 ± 0.31 a	7.87 ± 0.109a	7.17 ± 0.15 d	6.58 ± 0.06 c	13.92 ± 0.07 ab	20.20 ± 2.44 ab	32.88 ± 1.75 a
CFCR	17.18 ± 0.23 ab	6.21 ± 0.19 b	8.19 ± 0.10 c	6.50 ± 0.13 bc	14.18 ± 0.02 a	16.56 ± 3.95 ab	2.10 ± 0.29 d
CFLA	16.28 ± 0.20 b	6.63 ± 0.13 b	8.90 ± 0.22 b	7.12 ± 0.12 a	14.26 ± 0.09 a	20.00 ± 5.05 ab	25.14 ± 2.03 b

*^x^Colony characteristics: off white, with, off white mycelia with mass conidial masses.*

*^y^Data is the mean ± standard error. Mean values with the same letters were not statistically different (P > 0.05) according to the least significant difference (LSD) Test.*

*^z^CACR, C. aenigma causing crown rot; CSCR, C. siamense causing crown rot; CSLA, C. siamense causing leaf anthracnose; CFCR, C. fructicola causing crown rot; CFLA, C. fructicola causing leaf anthracnose.*

### Pathogenicity of *Colletotrichum* spp. Causing LA and CR

All the *Colletotrichum* spp. obtained from plants showing both LA and CR symptoms can cause leaf and crown disease when re-inoculated on strawberry, but the pathogenicity was significantly different (Supplementary Figure 1). Under wounded conditions, there were no significant differences in the pathogenicity of CSCR and CSLA to the leaves of “Hongjia” and “Hongyu.” The pathogenicity of CSLA was higher than that of CSCR to the leaves of “Zhangji”; the disease spot diameters caused by CSLA and CSCR were 13.39 and 11.85 mm, respectively. While in “Tianxianzui,” the pathogenicity of CSLA was weaker than that of CSCR, the disease spot diameters caused by CSLA and CSCR were 16.88 and 20.13 mm, respectively. Under non-wounded conditions, the pathogenicity of CSLA was weaker than that of CSCR in the “Zhangji” and “Hongyu” varieties (Table 4). For the crown, under wounded conditions, the pathogenicity of CSCR in different varieties was higher than that of CSLA (*P* < 0.05). Under non-wounded conditions, there was no significant difference in the pathogenicity of CSCR and CSLA (Table 5).

**TABLE 4:** Disease spot diameters (mm) of *Colletotrichum* species from leaf and crown of strawberry toward leaves of different strawberry varieties.

*Colletotrichum* spp.	Wounded condition				Non-wounded condition			
	Hongjia	Zhangji	Hongyu	Tianxianzui	Hongjia	Zhangji	Hongyu	Tianxianzui
CSCR^y^	13.92 ± 0.62 aB^x^	11.85 ± 0.39 bC	20.80 ± 1.44 aA	20.13 ± 0.67 aA	4.58 ± 0.29 bC	6.75 ± 0.29 aB	11.00 ± 0.98 aA	9.38 ± 0.81 aA
CSLA	12.73 ± 0.37 abC	13.39 ± 0.42 aC	18.96 ± 0.44 aA	16.88 ± 0.67 bB	3.81 ± 0.18 bB	4.21 ± 0.26 bcB	8.2 ± 0.63 bB	9.75 ± 0.54 aA
CFCR	11.33 ± 0.59 bA	7.75 ± 0.39 cC	9.42 ± 0.87 cB	12.06 ± 0.99 cA	6.00 ± 0.00 aA	3.25 ± 0.44 cB	3.50 ± 0.18 cB	3.5 ± 0.34 cB
CFLA	13.73 ± 0.50 aB	11.83 ± 0.46 bC	15.75 ± 0.77 bB	22.42 ± 1.60 aA	5.52 ± 0.51 bB	5.38 ± 0.20 bB	7.95 ± 0.62 bA	7.95 ± 0.68 bA

*^x^Data are mean ± standard error. Mean values with the same letters were not statistically different (P > 0.05) according to the least significant difference (LSD) test.*

*^y^CSCR, C. siamense causing crown rot; CSLA, C. siamense causing leaf anthracnose; CFCR, C. fructicola causing crown rot; CFLA, C. fructicola causing leaf anthracnose.*

**TABLE 5:** The disease index of *Colletotrichum* species from leaf and crown of strawberry toward crowns of different strawberry varieties.

*Colletotrichum* spp.	Wounded condition			Non-wounded condition		
	Hongjia	Zhangji	Tianxianzui	Hongjia	Zhangji	Tianxianzui
CSCR^y^	98.55 ± 5.68 abA^x^	41.92 ± 6.69 aC	82.08 ± 11.65 aB	49.22 ± 5.35 bA	12.11 ± 1.64 aB	25 ± 11.90 aB
CSLA	81.5 ± 4.96 bA	38.42 ± 4.43 abC	60.00 ± 5.19 abB	63.00 ± 2.88 abA	14.54 ± 0.81 aC	26.88 ± 3.26 aB
CFCR	95.00 ± 4.19 aA	30.73 ± 4.58 abC	55.78 ± 10.26 abB	70.00 ± 4.03 aA	13.1 ± 1.32 aB	18.17 ± 2.02 aB
CFLA	84.9 ± 5.54 abA	21.53 ± 1.83 bC	40.00 ± 6.71 bB	58.24 ± 3.79 abA	12.00 ± 1.22 aB	23.33 ± 4.77 aB

*^x^Data are mean ± standard error. Mean values with the same letters were not statistically different (P > 0.05) according to the least significant difference (LSD) test.*

*^y^CSCR, C. siamense causing crown rot; CSLA, C. siamense causing leaf anthracnose; CFCR, C. fructicola causing crown rot; CFLA, C. fructicola causing leaf anthracnose.*

CFLA and CFCR had significant differences in pathogenicity toward the leaves of the four varieties (Table 4). Under wounded and non-wounded conditions, the pathogenicity of CFLA to leaves was higher than that of CFCR (*P* < 0.01). For the crown, under wounded conditions, CFLA and CFCR showed significant differences in pathogenicity to the three varieties; the pathogenicity of CFCR on different varieties was higher than that of CFLA (Table 5). Under non-wounded conditions, there was no significant difference in the pathogenicity of CFCR and CFLA. CFCR and CFLA were the most pathogenic to “Hongjia,” followed by “Hongyu” and “Zhangji.”

## Discussion

Based on morphological and phylogenetic analysis, three species of *Colletotrichum*, *C. siamense*, *C. fructicola*, and *C. aenigma* were isolated and identified from strawberry plants showing both LA and CR symptoms in Zhejiang province, China. Among three identified species of *C*. *gloeosporioides* species complex, *C. siamense*, *C. fructicola* were associated with both leaf anthracnose and crown rot, while *C. aenigma* was only responsible for crown rot of strawberry plants. Previously, the *C*. *gloeosporioides* species complex has been reported to infect strawberries (Denoyes-Rothan et al., 2003), which is consist with the finding of Chen et al. (2020) that the *Colletotrichum* spp. on strawberries in Zhengjiang were all belonged to the *C*. *gloeosporioides* species complex, containing *C*. *fructicola*, *C*. *gloeosporioides*, *C*. *aenigma*, and *C*. *siamense*. However, *C*. *gloeosporioides* was not identified in our study, which may be due to the different sampling sites or the small sample base. Analysis of the samples collected in Spain showed that most of the *Colletotrichum* spp. that caused strawberry CR are *C*. *fragariae* and *C*. *acutatum* (MacKenzie et al., 2006), which often occur in warm and humid regions. The species causing LA are primarily *C*. *fragariae* and *C*. *gloeosporioides*, and *C*. *fragariae* is more pathogenic than *C*. *gloeosporioides* on the petiole (Ureña-Padilla et al., 2002). These results above indicated that the primary causing agents of LA and CR on strawberries are different. Consistently, in this study we speculated that the two diseases, LA and CR, might be caused by different pathogen sources and the species of pathogen were associated with the origin place of collection.

The morphological characterization showed that three *Colletotrichum* species differ greatly in growth rate, the size of spores and appressoria, sporulation, as well as spore germination rate, despite their similar colony morphology. The growth rate, sporulation, and spore germination rate of *C*. *siamense* was higher than those of *C*. *fructicola* and *C*. *aenigma*, which is consistent with Zhang et al. (2020). Chen et al. (2020) showed that sporulation of *C*. *fructicola* was the lowest, but the germination rate of spores was the highest, which was different from this study and may be due to reasons such as different host sources.

The pathogenicity results showed that *C*. *siamense* and *C*. *fructicola* can cause disease in the leaves and crown of different varieties, and their pathogenicity was significantly different among varieties. This may be related to disease resistance in the different strawberry varieties. The inoculation of *Colletotrichum* spp. onto the leaves and stolons of 12 varieties conducted by Wang et al. (2008) showed that “Hongjia” was a susceptible variety, and the incidence was more serious than that on “Fengxiang” and “Zhangji,” which was consistent with the results of this study. The results showed that the pathogenicity of *C*. *siamense* to leaves and the crown was higher than that of *C*. *fructicola*, which is also consistent with the results of Zhang et al. (2020). Under non-wounded conditions, the pathogenicity of *Colletotrichum* spp. to different strawberry varieties was relatively weak than that on wounded conditions (Jayawardena et al., 2016). This may occur because of the inefficient penetration of pathogens on the non-wounded plant cell wall (Than et al., 2008; Romero et al., 2018). Estrada et al. (2000) found that temperature and humidity can affect the pathogenicity, conidial germination, and appressoria formation of *Colletotrichum* spp., which raised our interests in further studying the impacts of temperature and humidity on the infection of *Colletotrichum* spp.

In this study, the phenotypic analysis of different *Colletotrichum* spp. showed that the metabolic rates of the three species on different nutrients were similar, but the metabolic capacity of different *Colletotrichum* species showed some differences. Among them, the nitrogen sources, nutritional supplement elements, and pH were significantly utilized by *C*. *siamense*. These substrates are widely found in plant tissues and thus affect pathogenicity (Wang et al., 2015, 2018). However, we did not compare the phenotype of crown and leaf isolates. Therefore, it is necessary to further study the relationship between *Colletotrichum* spp. diversity, metabolic phenotype, and pathogenicity.

We also analyzed the biological and pathogenicity characteristics of *Colletotrichum* spp. from strawberry plants showing both LA and CR symptoms. The sporulation of CFCR was less than that of CFLA, and CSCR was higher than CSLA. The spore germination rates of CSCR and CFCR were lower than those of CSLA and CFLA. The biological characteristics of the *Colletotrichum* spp. were related to the isolation part of strawberry plants. The spore germination rate may be related to virulence (Lin et al., 2012). Our results showed that CSLA, CFLA, CSCR, and CFCR can all infect the leaves and crown, but there was a significant difference (Turechek et al., 2002; Jayawardena et al., 2016). The pathogenicity of the isolates obtained from strawberry plants showing LA symptoms on leaves was higher than that on the crown, and the pathogenicity of CR isolates on the crown was higher than that on leaves. However, these results do not apply to all varieties and isolates. Curry et al. (2002) found that different *Colletotrichum* spp. infected strawberry with similar symptoms, but isolates from different isolation sites had differing pathogenicity. The isolate collected from the source part was more pathogenic at this part than at the non-source part, indicating that the isolate had a certain degree of tissue specialization. This was consistent with the results of Zhang and Xu (2005). Inoculating different parts of persimmon with *C*. *gloeosporioides* isolated from the fruit indicated that only the fruits and new branches were infected, while leaves were not. This indicates that the pathogenicity of pathogens from different sources had a certain degree of difference during the evolution process, and the level of disease resistance in the different strawberry varieties varied among the pathogens.

## Data Availability Statement

The original contributions presented in the study are included in the article/Supplementary Material, further inquiries can be directed to the corresponding author/s.

## Author Contributions

YZ, JW, and SH conceived the study. SH, JZ, and AL performed the computational analyses and interpreted the data. SH and MH prepared the figures and tables. CZ, SH, HY, and HW wrote and revised the manuscript. All authors helped to edit the manuscript and approved the final version.

## Conflict of Interest

The authors declare that the research was conducted in the absence of any commercial or financial relationships that could be construed as a potential conflict of interest.

## Publisher’s Note

All claims expressed in this article are solely those of the authors and do not necessarily represent those of their affiliated organizations, or those of the publisher, the editors and the reviewers. Any product that may be evaluated in this article, or claim that may be made by its manufacturer, is not guaranteed or endorsed by the publisher.

## Supplementary Material

Supplementary Figure 1 The pathogenicity of CSCR, CFCR, CSLA, CFLA to leaf and crown of different varieties. **(A)** The pathogenicity of CSCR, CFCR, CSLA, CFLA to leaf of Hongjia, Zhangji, Hongyu, Tianxianzui; W, wounded condition, UW, non-wounded condition. **(B)** Pathogenicity of CSCR, CFCR, CSLA, CFLA to crown of Hongjia, Zhangji, Tianxianzui under wounded condition.
