# Supplementary figures and images for: Colletotrichum Spp. Diversity Between Leaf Anthracnose and Crown Rot From the Same Strawberry Plant
Source: Front Microbiol. 2022 Apr 14;13:860694. doi: 10.3389/fmicb.2022.860694 (PMC9048825; doi:10.3389/fmicb.2022.860694)

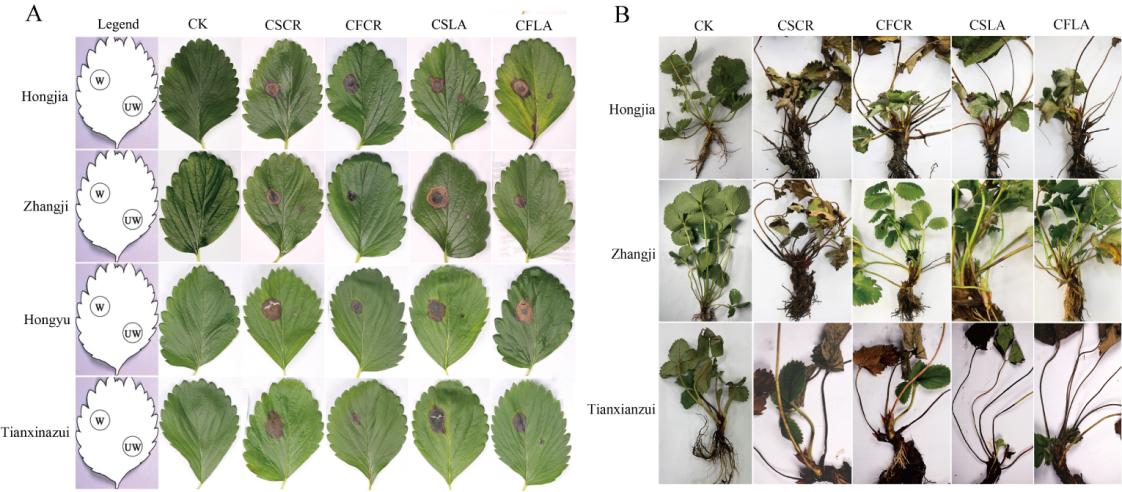

Supplement: Supplementary Figure 1 — The pathogenicity of CSCR, CFCR, CSLA, CFLA to leaf and crown of different varieties. (A) The pathogenicity of CSCR, CFCR, CSLA, CFLA to leaf of Hongjia, Zhangji, Hongyu, Tianxianzui; W, wounded condition, UW, non-wounded condition. (B) Pathogenicity of CSCR, CFCR, CSLA, CFLA to crown of Hongjia, Zhangji, Tianxianzui under wounded condition. [file Image_1.JPEG]
