# Supplementary material for: Colletotrichum Spp. Diversity Between Leaf Anthracnose and Crown Rot From the Same Strawberry Plant
Source: Front Microbiol. 2022 Apr 14;13:860694. doi: 10.3389/fmicb.2022.860694 (PMC9048825; doi:10.3389/fmicb.2022.860694)
Supplement: Supplementary file 2 [file Table_1.DOCX]

^TABLE S1^ *^Colletotrichum^* ^spp. used in multi-gene analysis in this study.^

| **Species** | **Isolates ^1^** | **Host/substrate** | **Varities** | **Country** | **GenBank no.** | | | | |
| --- | --- | --- | --- | --- | --- | --- | --- | --- | --- |
|  |  |  |  |  | **ITS** | **ACT** | **CAL** | **CHS** | **GAPDH** |
| *C. aenigma* | ICMP 18686^*^ | *Pyrus pyrifolia* |  | Japan | JX010243 | JX009519 | JX009684 | JX009789 | JX009913 |
|  | JD-HX-A-21 | *Fragaria × ananassa*, Crown | Hongjia | Jiande, Zhejiang, China | MW513755 | MW513845 | MW513893 | MW513869 | MW513917 |
| *C.aeschynomenes* | ICMP 17673^*^ | *Aeschynomene virginica* |  | USA | JX010176 | JX009483 | JX009721 | JX009799 | JX009930 |
| *C. alatae* | ICMP 17919^*^ | *Dioscorea alata* |  | India | JX010190 | JX009471 | JX009738 | JX009837 | JX009990 |
| *C. alienum* | ICMP 12071^*^ | *Malus domestica* |  | New Zealand | JX010251 | JX009572 | JX009654 | JX009882 | JX010028 |
| *C. aotearoa* | ICMP 18537^*^ | *Coprosma* sp. |  | New Zealand | JX010205 | JX009564 | JX009611 | JX009853 | JX010005 |
| *C.asianum* | ICMP 18580^*^ | *Coffea arabica* |  | Thailand | FJ972612 | JX009584 | FJ917506 | JX009867 | JX010053 |
| *C. boninense* | CBS 123755^*^ | *Crinum asiaticum var. sinicum* |  | Japan | JX010292 | JX009583 | JQ005674 | JX009827 | JX009905 |
| *C. clidemiae* | ICMP 18706^*^ | *Clidemia hirta* |  | USA | JX010274 | JX009476 | JX009639 | JX009777 | JX009909 |
| *C. cordylinicola* | ICMP 18579^*^ | *Cordyline fruticosa* |  | Thailand | JX010226 | HM470235 | HM470238 | JX009864 | JX009975 |
| *C. endophytica* | CAUG28 | *Capsicum annuum* |  | China | KP145441 | KP145329 | KP145357 | KP145385 | KP145413 |
| *C.fructicola* | ICMP 18581^*^ | *Coffea arabica* |  | Thailand | JX010165 | FJ907426 | FJ917508 | JX009866 | JX010033 |
|  | CBS 120005^*^ | *Fragaria × ananassa* |  | USA | JX010175 | JX009534 | JX009673 | JX009792 | JX009926 |
|  | JD-HX-Y-H-22 | *Fragaria × ananassa*, Leaf | Hongjia | Jiande, Zhejiang, China | MW647704 | MW695953 | MW696004 | MW696055 | MW696106 |
|  | JD-ZJ-Y-H-69 | *Fragaria × ananassa*, Leaf | Zhangji | Jiande, Zhejiang, China | MW647705 | MW695954 | MW696005 | MW696056 | MW696107 |
|  | ZS-ZJ-Y-2-1 | *Fragaria × ananassa*, Leaf | Zhangji | Zhoushan, Zhejiang, China | MW647706 | MW695955 | MW696006 | MW696057 | MW696108 |
|  | ZS-ZJ-Y-2-2 | *Fragaria × ananassa*, Leaf | Zhangji | Zhoushan, Zhejiang, China | MW647707 | MW695956 | MW696007 | MW696058 | MW696109 |
|  | JD-ZJ-3-2 | *Fragaria × ananassa*, Crown | Zhangji | Jiande, Zhejiang, China | MW513775 | MW513825 | MW513829 | MW513833 | MW513837 |
|  | JD-ZJ-5 | *Fragaria × ananassa*, Crown | Zhangji | Jiande, Zhejiang, China | MW513776 | MW513826 | MW513830 | MW513834 | MW513838 |
|  | JD-ZJ-9 | *Fragaria × ananassa*, Crown | Zhangji | Jiande, Zhejiang, China | MW513777 | MW513827 | MW513831 | MW513835 | MW513839 |
|  | JD-ZJ-12 | *Fragaria × ananassa*, Crown | Zhangji | Jiande, Zhejiang, China | MW513778 | MW513828 | MW513832 | MW513836 | MW513840 |
| *C. gloeosporiodes* | IMI 356878^*^ | *Citrus sinensis* |  | Italy | JX010152 | JX009531 | JX009731 | JX009818 | JX010056 |
| *C. horri* | ICMP 10492^*^ | *Diospyros kaki* |  | Japan | GQ329690 | JX009438 | JX009604 | JX009752 | GQ329681 |
| *C. kahawae* | ICMP 17816^*^ | *Coffea arabica* |  | Kenya | JX010231 | JX009452 | JX009642 | JX009813 | JX010012 |
| *C. musae* | ICMP 19119^*^ | *Musa* sp. |  | USA | JX010146 | JX009433 | JX009742 | JX009896 | JX010050 |
| *C. nupharicola* | CBS 470.96^*^ | *Nuphar lutea* subsp. *polysepala* |  | USA | JX010187 | JX009437 | JX009663 | JX009835 | JX009972 |
| *C. proteae* | CBS 132882^*^ | *Protea* sp. |  | South Africa | KC297079 | KC296940 | KC296960 | KC297101 | KC297009 |
| *C. psidii* | CBS 145.29^*^ | *Psidium* sp. |  | Italy | JX010219 | JX009515 | JX009743 | JX009901 | JX009967 |
| *C. queenslandicum* | ICMP 1778^*^ | *Carica papaya* |  | Australia | JX010276 | JX009447 | JX009691 | JX009899 | JX009934 |
| *C. salsolae* | ICMP 19051^*^ | *Salsola tragus* |  | Hungary | JX010242 | JX009562 | JX009696 | JX009863 | JX009916 |
| *C.siamense* | ICMP 17785^*^ | *Malus domestica* |  | USA | JX010272 | JX009446 | JX009706 | JX009804 | JX010051 |
|  | ICMP 18572^*^ | *Vitis vinifera* |  | USA | JX010160 | JX009487 | JX009705 | JX009783 | JX010061 |
|  | JD-HX-Y-B-7 | *Fragaria × ananassa*, Leaf | Hongjia | Jiande, Zhejiang, China | MW647796 | MW695957 | MW696008 | MW696059 | MW696110 |
|  | JD-HX-Y-B-16 | *Fragaria × ananassa*, Leaf | Hongjia | Jiande, Zhejiang, China | MW647797 | MW695958 | MW696009 | MW696060 | MW696111 |
|  | JD-HX-Y-B-11 | *Fragaria × ananassa*, Leaf | Hongjia | Jiande, Zhejiang, China | MW647798 | MW695959 | MW696010 | MW696061 | MW696112 |
|  | JD-HX-Y-B-18 | *Fragaria × ananassa*, Leaf | Hongjia | Jiande, Zhejiang, China | MW647799 | MW695960 | MW696011 | MW696062 | MW696113 |
|  | JD-HX-Y-H-4 | *Fragaria × ananassa*, Leaf | Hongjia | Jiande, Zhejiang, China | MW647800 | MW695961 | MW696012 | MW696063 | MW696114 |
|  | JD-HX-Y-H-11 | *Fragaria × ananassa*, Leaf | Hongjia | Jiande, Zhejiang, China | MW647801 | MW695962 | MW696013 | MW696064 | MW696115 |
|  | JD-HX-Y-H-16 | *Fragaria × ananassa*, Leaf | Hongjia | Jiande, Zhejiang, China | MW647802 | MW695963 | MW696014 | MW696065 | MW696116 |
|  | JD-HX-Y-H-20 | *Fragaria × ananassa*, Leaf | Hongjia | Jiande, Zhejiang, China | MW647803 | MW695964 | MW696015 | MW696066 | MW696117 |
|  | JD-HX-Y-H-23 | *Fragaria × ananassa*, Leaf | Hongjia | Jiande, Zhejiang, China | MW647804 | MW695965 | MW696016 | MW696067 | MW696118 |
|  | JD-TXZ-Y-1 | *Fragaria × ananassa*, Leaf | Tianxianzui | Jiande, Zhejiang, China | MW647805 | MW695966 | MW696017 | MW696068 | MW696119 |
|  | JD-TXZ-Y-2 | *Fragaria × ananassa*, Leaf | Tianxianzui | Jiande, Zhejiang, China | MW647806 | MW695967 | MW696018 | MW696069 | MW696120 |
|  | JD-TXZ-Y-3 | *Fragaria × ananassa*, Leaf | Tianxianzui | Jiande, Zhejiang, China | MW647807 | MW695968 | MW696019 | MW696070 | MW696121 |
|  | JD-TXZ-Y-4 | *Fragaria × ananassa*, Leaf | Tianxianzui | Jiande, Zhejiang, China | MW647808 | MW695969 | MW696020 | MW696071 | MW696122 |
|  | JD-TXZ-Y-5 | *Fragaria × ananassa*, Leaf | Tianxianzui | Jiande, Zhejiang, China | MW647809 | MW695970 | MW696021 | MW696072 | MW696123 |
|  | JD-TXZ-Y-6 | *Fragaria × ananassa*, Leaf | Tianxianzui | Jiande, Zhejiang, China | MW647810 | MW695971 | MW696022 | MW696073 | MW696124 |
|  | JD-TXZ-Y-7 | *Fragaria × ananassa*, Leaf | Tianxianzui | Jiande, Zhejiang, China | MW647811 | MW695972 | MW696023 | MW696074 | MW696125 |
|  | JD-TXZ-Y-8 | *Fragaria × ananassa*, Leaf | Tianxianzui | Jiande, Zhejiang, China | MW647812 | MW695973 | MW696024 | MW696075 | MW696126 |
|  | JD-TXZ-Y-9 | *Fragaria × ananassa*, Leaf | Tianxianzui | Jiande, Zhejiang, China | MW647813 | MW695974 | MW696025 | MW696076 | MW696127 |
|  | JD-ZJ-Y-B-6 | *Fragaria × ananassa*, Leaf | Zhangji | Jiande, Zhejiang, China | MW647814 | MW695975 | MW696026 | MW696077 | MW696128 |
|  | JD-ZJ-Y-H-2 | *Fragaria × ananassa*, Leaf | Zhangji | Jiande, Zhejiang, China | MW647815 | MW695976 | MW696027 | MW696078 | MW696129 |
|  | JD-ZJ-Y-H-6 | *Fragaria × ananassa*, Leaf | Zhangji | Jiande, Zhejiang, China | MW647816 | MW695977 | MW696028 | MW696079 | MW696130 |
|  | JD-ZJ-Y-H-11 | *Fragaria × ananassa*, Leaf | Zhangji | Jiande, Zhejiang, China | MW647817 | MW695978 | MW696029 | MW696080 | MW696131 |
|  | JD-ZJ-Y-H-15 | *Fragaria × ananassa*, Leaf | Zhangji | Jiande, Zhejiang, China | MW647818 | MW695979 | MW696030 | MW696081 | MW696132 |
|  | JD-ZJ-Y-H-60 | *Fragaria × ananassa*, Leaf | Zhangji | Jiande, Zhejiang, China | MW647819 | MW695980 | MW696031 | MW696082 | MW696133 |
|  | JD-ZJ-Y-H-64 | *Fragaria × ananassa*, Leaf | Zhangji | Jiande, Zhejiang, China | MW647820 | MW695981 | MW696032 | MW696083 | MW696134 |
|  | JD-ZJ-Y-H-74 | *Fragaria × ananassa*, Leaf | Zhangji | Jiande, Zhejiang, China | MW647821 | MW695982 | MW696033 | MW696084 | MW696135 |
|  | JD-ZJ-Y-H-77 | *Fragaria × ananassa*, Leaf | Zhangji | Jiande, Zhejiang, China | MW647822 | MW695983 | MW696034 | MW696085 | MW696136 |
|  | ZS-HX-Y-2 | *Fragaria × ananassa*, Leaf | Hongjia | Zhoushan, Zhejiang, China | MW647824 | MW695985 | MW696036 | MW696087 | MW696138 |
|  | ZS-HX-Y-3 | *Fragaria × ananassa*, Leaf | Hongjia | Zhoushan, Zhejiang, China | MW647825 | MW695986 | MW696037 | MW696088 | MW696139 |
|  | ZS-HX-Y-4 | *Fragaria × ananassa*, Leaf | Hongjia | Zhoushan, Zhejiang, China | MW647826 | MW695987 | MW696038 | MW696089 | MW696140 |
|  | ZS-HX-Y-5 | *Fragaria × ananassa*, Leaf | Hongjia | Zhoushan, Zhejiang, China | MW647827 | MW695988 | MW696039 | MW696090 | MW696141 |
|  | ZS-HX-Y-6 | *Fragaria × ananassa*, Leaf | Hongjia | Zhoushan, Zhejiang, China | MW647828 | MW695989 | MW696040 | MW696091 | MW696142 |
|  | ZS-HX-Y-7 | *Fragaria × ananassa*, Leaf | Hongjia | Zhoushan, Zhejiang, China | MW647829 | MW695990 | MW696041 | MW696092 | MW696143 |
|  | ZS-HX-Y-8 | *Fragaria × ananassa*, Leaf | Hongjia | Zhoushan, Zhejiang, China | MW647830 | MW695991 | MW696042 | MW696093 | MW696144 |
|  | ZS-HX-Y-9 | *Fragaria × ananassa*, Leaf | Hongjia | Zhoushan, Zhejiang, China | MW647831 | MW695992 | MW696043 | MW696094 | MW696145 |
|  | ZS-HX-Y-26 | *Fragaria × ananassa*, Leaf | Hongjia | Zhoushan, Zhejiang, China | MW647832 | MW695993 | MW696044 | MW696095 | MW696146 |
|  | ZS-HX-Y-33 | *Fragaria × ananassa*, Leaf | Hongjia | Zhoushan, Zhejiang, China | MW647833 | MW695994 | MW696045 | MW696096 | MW696147 |
|  | ZS-HX-Y-39 | *Fragaria × ananassa*, Leaf | Hongjia | Zhoushan, Zhejiang, China | MW647834 | MW695995 | MW696046 | MW696097 | MW696148 |
|  | ZS-ZJ-Y-1-1 | *Fragaria × ananassa*, Leaf | Zhangji | Zhoushan, Zhejiang, China | MW647835 | MW695996 | MW696047 | MW696098 | MW696149 |
|  | ZS-ZJ-Y-1-2 | *Fragaria × ananassa*, Leaf | Zhangji | Zhoushan, Zhejiang, China | MW647836 | MW695997 | MW696048 | MW696099 | MW696150 |
|  | ZS-ZJ-Y-41 | *Fragaria × ananassa*, Leaf | Zhangji | Zhoushan, Zhejiang, China | MW647837 | MW695998 | MW696049 | MW696100 | MW696151 |
|  | ZS-ZJ-Y-44 | *Fragaria × ananassa*, Leaf | Zhangji | Zhoushan, Zhejiang, China | MW647838 | MW695999 | MW696050 | MW696101 | MW696152 |
|  | ZS-ZJ-Y-47 | *Fragaria × ananassa*, Leaf | Zhangji | Zhoushan, Zhejiang, China | MW647839 | MW696000 | MW696051 | MW696102 | MW696153 |
|  | ZS-ZJ-Y-50 | *Fragaria × ananassa*, Leaf | Zhangji | Zhoushan, Zhejiang, China | MW647840 | MW696001 | MW696052 | MW696103 | MW696154 |
|  | ZS-ZJ-Y-53 | *Fragaria × ananassa*, Leaf | Zhangji | Zhoushan, Zhejiang, China | MW647841 | MW696002 | MW696053 | MW696104 | MW696155 |
|  | ZS-ZJ-Y-56 | *Fragaria × ananassa*, Leaf | Zhangji | Zhoushan, Zhejiang, China | MW647842 | MW696003 | MW696054 | MW696105 | MW696156 |
|  | ZS-HX-54 | *Fragaria × ananassa*, Crown | Hongjia | Zhoushan, Zhejiang, China | MW513762 | MW513852 | MW513900 | MW513876 | MW513924 |
|  | ZS-HX-77 | *Fragaria × ananassa*, Crown | Hongjia | Zhoushan, Zhejiang, China | MW513764 | MW513854 | MW513902 | MW513878 | MW513926 |
|  | ZS-HX-85 | *Fragaria × ananassa*, Crown | Hongjia | Zhoushan, Zhejiang, China | MW513765 | MW513855 | MW513903 | MW513879 | MW513927 |
|  | ZS-HX-111 | *Fragaria × ananassa*, Crown | Hongjia | Zhoushan, Zhejiang, China | MW513766 | MW513856 | MW513904 | MW513880 | MW513928 |
|  | ZS-ZJ-6 | *Fragaria × ananassa*, Crown | Zhangji | Zhoushan, Zhejiang, China | MW513767 | MW513857 | MW513905 | MW513881 | MW513929 |
|  | ZS-ZJ-20 | *Fragaria × ananassa*, Crown | Zhangji | Zhoushan, Zhejiang, China | MW513768 | MW513858 | MW513906 | MW513882 | MW513930 |
|  | ZS-ZJ-32 | *Fragaria × ananassa*, Crown | Zhangji | Zhoushan, Zhejiang, China | MW513769 | MW513859 | MW513907 | MW513883 | MW513931 |
|  | ZS-ZJ-49 | *Fragaria × ananassa*, Crown | Zhangji | Zhoushan, Zhejiang, China | MW513770 | MW513860 | MW513908 | MW513884 | MW513932 |
|  | ZS-ZJ-53 | *Fragaria × ananassa*, Crown | Zhangji | Zhoushan, Zhejiang, China | MW513771 | MW513861 | MW513909 | MW513885 | MW513933 |
|  | JD-HX-A-3 | *Fragaria × ananassa*, Crown | Hongjia | Jiande, Zhejiang, China | MW513751 | MW513841 | MW513889 | MW513865 | MW513913 |
|  | JD-HX-A-6 | *Fragaria × ananassa*, Crown | Hongjia | Jiande, Zhejiang, China | MW513752 | MW513842 | MW513890 | MW513866 | MW513914 |
|  | JD-HX-A-12 | *Fragaria × ananassa*, Crown | Hongjia | Jiande, Zhejiang, China | MW513753 | MW513843 | MW513891 | MW513867 | MW513915 |
|  | JD-HX-A-16 | *Fragaria × ananassa*, Crown | Hongjia | Jiande, Zhejiang, China | MW513754 | MW513844 | MW513892 | MW513868 | MW513916 |
|  | JD-ZJ-16 | *Fragaria × ananassa*, Crown | Zhangji | Jiande, Zhejiang, China | MW513761 | MW513851 | MW513899 | MW513870 | MW513923 |
|  | JD-TXZ-2 | *Fragaria × ananassa*, Crown | Tianxianzui | Jiande, Zhejiang, China | MW513756 | MW513846 | MW513894 | MW513871 | MW513918 |
|  | JD-TXZ-7 | *Fragaria × ananassa*, Crown | Tianxianzui | Jiande, Zhejiang, China | MW513757 | MW513847 | MW513895 | MW513872 | MW513919 |
|  | JD-TXZ-12 | *Fragaria × ananassa*, Crown | Tianxianzui | Jiande, Zhejiang, China | MW513758 | MW513848 | MW513896 | MW513873 | MW513920 |
|  | JD-TXZ-15 | *Fragaria × ananassa*, Crown | Tianxianzui | Jiande, Zhejiang, China | MW513759 | MW513849 | MW513897 | MW513874 | MW513921 |
|  | JD-TXZ-18 | *Fragaria × ananassa*, Crown | Tianxianzui | Jiande, Zhejiang, China | MW513760 | MW513850 | MW513898 | MW513875 | MW513922 |
|  | JD-HX-J-A-1 | *Fragaria × ananassa*, Crown | Hongjia | Jiande, Zhejiang, China | MW653754 | MW695845 | MW695872 | MW695899 | MW695926 |
|  | JD-HX-J-A-9 | *Fragaria × ananassa*, Crown | Hongjia | Jiande, Zhejiang, China | MW653755 | MW695846 | MW695873 | MW695900 | MW695927 |
|  | JD-HX-J-A-19 | *Fragaria × ananassa*, Crown | Hongjia | Jiande, Zhejiang, China | MW653756 | MW695847 | MW695874 | MW695901 | MW695928 |
|  | JD-HX-J-A-23 | *Fragaria × ananassa*, Crown | Hongjia | Jiande, Zhejiang, China | MW653757 | MW695848 | MW695875 | MW695902 | MW695929 |
|  | JD-HX-J-A-28 | *Fragaria × ananassa*, Crown | Hongjia | Jiande, Zhejiang, China | MW653758 | MW695849 | MW695876 | MW695903 | MW695930 |
|  | JD-TXZ-J-1 | *Fragaria × ananassa*, Crown | Tianxianzui | Jiande, Zhejiang, China | MW653759 | MW695850 | MW695877 | MW695904 | MW695931 |
|  | JD-TXZ-J-6 | *Fragaria × ananassa*, Crown | Tianxianzui | Jiande, Zhejiang, China | MW653760 | MW695851 | MW695878 | MW695905 | MW695932 |
|  | JD-TXZ-J-11 | *Fragaria × ananassa*, Crown | Tianxianzui | Jiande, Zhejiang, China | MW653761 | MW695852 | MW695879 | MW695906 | MW695933 |
|  | JD-TXZ-J-16 | *Fragaria × ananassa*, Crown | Tianxianzui | Jiande, Zhejiang, China | MW653762 | MW695853 | MW695880 | MW695907 | MW695934 |
|  | JD-ZJ-J-2-2 | *Fragaria × ananassa*, Crown | Zhangji | Jiande, Zhejiang, China | MW653763 | MW695854 | MW695881 | MW695908 | MW695935 |
|  | JD-ZJ-J-7 | *Fragaria × ananassa*, Crown | Zhangji | Jiande, Zhejiang, China | MW653764 | MW695855 | MW695882 | MW695909 | MW695936 |
|  | JD-ZJ-J-10 | *Fragaria × ananassa*, Crown | Zhangji | Jiande, Zhejiang, China | MW653765 | MW695856 | MW695883 | MW695910 | MW695937 |
|  | JD-ZJ-J-11 | *Fragaria × ananassa*, Crown | Zhangji | Jiande, Zhejiang, China | MW653766 | MW695857 | MW695884 | MW695911 | MW695938 |
|  | JD-ZJ-J-B-7 | *Fragaria × ananassa*, Crown | Zhangji | Jiande, Zhejiang, China | MW653767 | MW695858 | MW695885 | MW695912 | MW695939 |
|  | ZS-HX-J-59 | *Fragaria × ananassa*, Crown | Hongjia | Zhoushan, Zhejiang, China | MW653768 | MW695859 | MW695886 | MW695913 | MW695940 |
|  | ZS-HX-J-70 | *Fragaria × ananassa*, Crown | Hongjia | Zhoushan, Zhejiang, China | MW653769 | MW695860 | MW695887 | MW695914 | MW695941 |
|  | ZS-HX-J-93 | *Fragaria × ananassa*, Crown | Hongjia | Zhoushan, Zhejiang, China | MW653770 | MW695861 | MW695888 | MW695915 | MW695942 |
|  | ZS-HX-J-99 | *Fragaria × ananassa*, Crown | Hongjia | Zhoushan, Zhejiang, China | MW653771 | MW695862 | MW695889 | MW695916 | MW695943 |
|  | ZS-HX-J-114 | *Fragaria × ananassa*, Crown | Hongjia | Zhoushan, Zhejiang, China | MW653772 | MW695863 | MW695890 | MW695917 | MW695944 |
|  | ZS-HX-J-116 | *Fragaria × ananassa*, Crown | Hongjia | Zhoushan, Zhejiang, China | MW653773 | MW695864 | MW695891 | MW695918 | MW695945 |
|  | ZS-HX-J-119 | *Fragaria × ananassa*, Crown | Zhangji | Zhoushan, Zhejiang, China | MW653774 | MW695865 | MW695892 | MW695919 | MW695946 |
|  | ZS-ZJ-J-15 | *Fragaria × ananassa*, Crown | Zhangji | Zhoushan, Zhejiang, China | MW653776 | MW695867 | MW695894 | MW695921 | MW695948 |
|  | ZS-ZJ-J-26 | *Fragaria × ananassa*, Crown | Zhangji | Zhoushan, Zhejiang, China | MW653777 | MW695868 | MW695895 | MW695922 | MW695949 |
|  | ZS-ZJ-J-36 | *Fragaria × ananassa*, Crown | Zhangji | Zhoushan, Zhejiang, China | MW653778 | MW695869 | MW695896 | MW695923 | MW695950 |
|  | ZS-ZJ-J-42 | *Fragaria × ananassa*, Crown | Zhangji | Zhoushan, Zhejiang, China | MW653779 | MW695870 | MW695897 | MW695924 | MW695951 |
|  | ZS-ZJ-J-45 | *Fragaria × ananassa*, Crown | Zhangji | Zhoushan, Zhejiang, China | MW653780 | MW695871 | MW695898 | MW695925 | MW695952 |
| *C. theobromicola* | ICMP 18649^*^ | *Theobroma cacao* |  | Panama | JX010294 | JX009444 | JX009591 | JX009869 | JX010006 |
| *C. ti* | ICMP 4832^*^ | *Cordyline* sp. |  | New Zealand | JX010269 | JX009520 | JX009649 | JX009898 | JX009952 |
| *C.tropicale* | ICMP 18653^*^ | *Theobroma cacao* |  | Panama | JX010264 | JX009489 | JX009719 | JX009870 | JX010007 |
| *C. viniferum* | CAUG27 | *Capsicum* sp. |  | China | KP145440 | KP145328 | KP145356 | KP145384 | KP145412 |
| *C. xanthorrhoeae* | ICMP 17903^*^ | *Xanthorrhoea preissii* |  | Australia | JX010261 | JX009478 | JX009653 | JX009823 | JX009927 |
